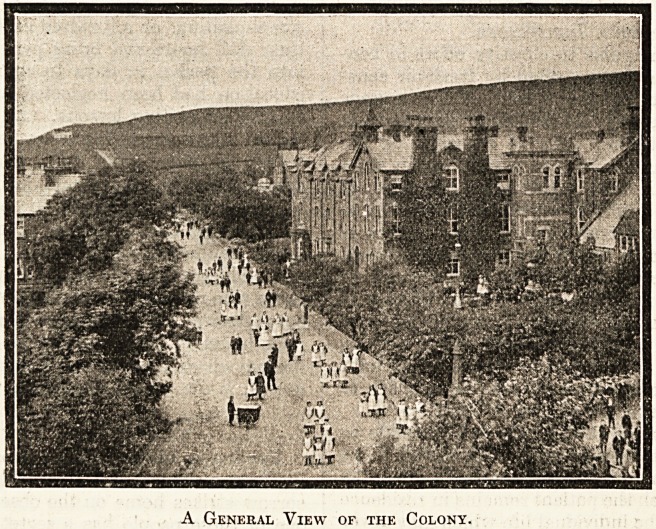# The National Children's Home at Edgworth, Lancashire

**Published:** 1912-04-13

**Authors:** Conrad W. Thies

**Affiliations:** late Secretary Royal Free Hospital.


### The National Children's Home at Edgworth, Lancashire.

By CONRAD W. THIES, late Secretary Royal Free Hospital.

After attending the Second Annual Conference of the British Hospitals Association last autumn in Manchester, I was glad of the opportunity to revisit the Lancashire Branch of the National Children's Home, which is situated on the moors at Edgworth, a few miles from Bolton. After visiting hospitals where disease is treated it is instructive to pass on to the convalescent and constructive side of institutional work, a side which in hospital circles is sometimes not remembered enough.

It was interesting to recall my first visit in 1873, when I accompanied the first party of young emigrants sent from the National Children's Home to Canada; but a remarkable change, and development has taken place during the intervening years.

There formerly stood upon this spot a lonely wayside inn called "The Wheatsheaf," which had long been notorious as the resort of the rowdiest characters of Bolton and the neighbourhood, who came thither to indulge in drinking, cock-fighting, dog-fighting, and other similar sports, so that in spite of the efforts of the police, the inn had become a public nuisance. In 1872, the late Mr. James Barlow of Bolton, the father of Sir Thomas Barlow, bought the inn with some eighty acres of the surrounding rough moorland, which has since been increased to 200 acres. Mr. Barlow extinguished the licence of the public-house and generously presented the property as a free gift to the National Children's Home, which had been founded in London a few years previously by the Rev. Dr. Bowman Stephenson for the succour and training of poor children.

The old Wheatsheaf Inn, which was a substantial grey stone building, was at once adapted for the reception of twenty boys. It still stands as the centre of the village, which now comprises twenty large houses, each accommodating a family of some twenty children with a Sister in Charge; there are also a church, schools, workshops, laundry, dairy and farm buildings, with a population of 320 children, besides the necessary staff of officers.

When I recalled how Mr. Barlow had driven me round the estate soon after its purchase and the hopes that he then expressed of its future usefulness to poor children, I could not help reflecting upon the vast amount of good that has resulted from his gift.

The enterprise of turning this wild stretch of uncultivated moorland into fruitful fields, and these apparently predestined hospital cases into healthy young men and women was no light task, but fortunately the right man was found in Mr. Alfred W. Mager, who sacrificed a good commercial position in London to devote himself to the work, and after thirty-five years of the most strenuous toil, in which he was ably assisted by his wife, has recently retired. It must be a source of much gratification to Mr. and Mrs. Mager to know that such a large number of boys and girls from the great Lancashire towns and from all parts of the kingdom have been saved from ill-health and trained under their care to become productive members of society.

The present Governor, Mr. Walter H. Wadhams, worked with Mr. Mager for some years and now carries on the responsible supervision of this busy colony of young folk. In his company I visited many of the houses, workshops, etc. I saw the boys and girls both at work and at play, I found them busily employed in carpenters' and cobblers' shops, in the smithy, the laundry, the dairy, and the bakehouse. The place, indeed, is a hive of industry, where the young people seem to produce nearly everything for their own requirements. I visited the farm buildings, I saw where they had quarried the stone of which the buildings and walls have been built, how they had assisted in making a large reservoir, and in carrying out elaborate schemes of levelling and drainage.

As a previous visitor to the Institution has well said: "What would become of many of these destitute little ones under ordinary circumstances? They would leave, or be cast out of their dreary dens of homes: they would drift into squalor, misery, and crime: they would die early or become public encumbrances in hospital, or workhouse." The Home has made its occupants first healthy, then efficient, and therefore employable. It is the topmost rung of that steep ladder in which the hospital and the convalescent home are the lowest and middle grades.

The Edgworth Home is only one branch of the National Children's Home and it may now claim to be one of the largest child-saving institutions in the world. It at present shelters some 2,250 children and more than 10,000 have already been trained and started in life. The headquarters are at Bonner Road, London, and, beside the Farm Colony at Edgworth, there are a beautiful children's hospital and convalescent home at Chipping Norton; together with an institution for treating delicate children at Alverstoke. The Canadian Branch is situated at Hamilton, Ont., and serves both as a distributing home and general headquarters of the Canadian work. Some 2,800 boys and girls have already been sent to Canada, the majority of whom are doing well.

An estate of 200 acres has recently been acquired at Harpenden, Herts, where a sanatorium for fifty children threatened with consumption has already been opened. It is hoped to transfer the present London establishment to this estate within the next twelve months.

A General View of the Colony.

It costs only £16 a year to train a neglected child in these homes, whereas the country spends many pounds annually upon each of its Poor Law infirmary cases, and £80 on each of its criminals. It is because the future must lie with preventive medicine and public health of all sorts that this account of saved material is so interesting to hospital men. By the institution of almoners, the voluntary hospitals have tentatively begun that constructive work of which this home is the complete phase. The work of the almoners will grow ever less detective and more helpful to patients, but the struggle against disease and degeneracy will not be complete till there has grown up a close cooperation between the hospitals and such homes as these. Then we may confidently hope that in time our country may be enriched and ennobled by the very classes which are now its danger and disgrace.

## Figures and Tables

**Figure f1:**